# Projectile Penetration into Calcareous Sand Subgrade Airport Runway Pavement with Genetic Algorithm Optimization

**DOI:** 10.3390/ma17235696

**Published:** 2024-11-21

**Authors:** Chucai Peng, Jingnan Huang, Xichen Sun, Yifei Nan, Yaohui Chen, Kun Chen, Jun Feng

**Affiliations:** 1 School of Civil Engineering and Architecture, Hunan Institute of Science and Technology, Yueyang 414006, China; 12017016@hnist.edu.cn; 2 National Key Laboratory of Transient Physics, Nanjing University of Science and Technology, Nanjing 210094, China; yhchen@njust.edu.cn (Y.C.); 12016120@njust.edu.cn (K.C.); 3 School of Civil and Environmental Engineering, University of New South Wales, Sydney 2052, Australia; ahhsgxxn@163.com; 4 School of Astronautics, Harbin Institute of Technology, Harbin 150001, China; 23s018004@stu.hit.edu.cn

**Keywords:** reef island runways, discrete element method, pavement structure penetration, genetic algorithm, critical striking velocity

## Abstract

As an important civil and military infrastructure, airport runway pavement is faced with threats from cluster munitions, since it is vulnerable to projectile impacts with internal explosions. Aiming at the damage assessment of an island airport runway pavement under impact, this work dealt with discrete modeling of rigid projectile penetration into concrete pavement and the calcareous sand subgrade multi-layer structure. First, the Discrete Element Method (DEM) is introduced to model concrete and calcareous sand granular material features, like cohesive fracture and strain hardening due to compression, with mesoscale constitutive laws governing the normal and shear interactions between adjacent particles. Second, the subsequent DEM simulations of uniaxial and triaxial compression were performed to calibrate the DEM parameters for pavement concrete, as well as subgrade calcareous sand. Prior to the multi-layer structure investigations, penetration into sole concrete or calcareous sand is validated in terms of projectile deceleration and depth of penetration (DOP) with relative error ≤ 5.6% providing a reliable numerical tool for deep penetration damage assessments. Third, projectile penetration into the airport runway structure with concrete pavement and calcareous sand subgrade was evaluated with validated DEM model. Penetration numerical simulations with various projectile weight, pavement concrete thickness as well as striking velocity, were performed to achieve the DOP. Moreover, the back-propagation (BP) neural network proxy model was constructed to predict the airport runway penetration data with good agreement realizing rapid and robust DOP forecasting. Finally, the genetic algorithm was coupled with the proxy model to realize intelligent optimization of pavement penetration, whereby the critical velocity projectile just perforates concrete pavement indicating the severest subsequent munition explosion damage.

## 1. Introduction

Airports have been hot targets of military attacks owing to their large footprint, obvious targets, and prominent military status, which can effectively neutralize the enemy’s capability to conduct air operations [1]. Mainly composed of a surface layer, a base layer, and a compacted soil layer, the airport runway is a typical layered structure [2]. Although some studies proved that asphalt concrete surfaces could absorb the impact energy [3], the concrete pavements is widely used in the airports due to the fact that the high temperature and aircraft load generated by takeoffs of jet airplanes can damage and deteriorate asphalt surfaces [4].

**Figure 1:**
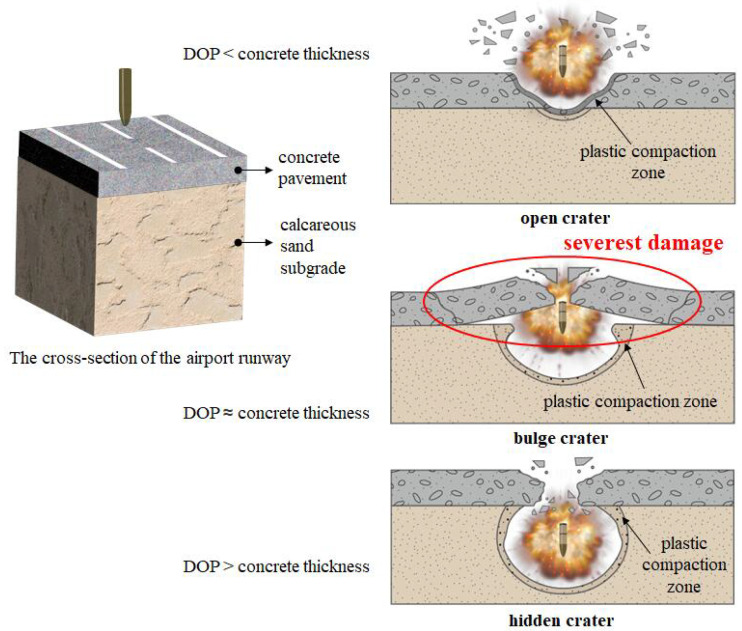
Island airport runway under cluster munitions attack.

Calcareous sand is a sediment primarily composed of the skeletal remains of marine organisms [5], with calcium carbonate accounting for over 97% of its weight [6]. It is widely found in the coral reefs of the Nansha Islands in the South China Sea, and is characterized by high intragranular porosity [7], significant particle angularity [8], irregular shape, and a tendency to break easily [9]. Moreover, calcareous sand exhibits higher apparent cohesion than quartz sand, and demonstrates considerable load-bearing capacity after compaction [10], making it an ideal backfilling material for the subgrade of airport runways in marine engineering. Additionally, due to the remote location of the South China Sea islands, using calcareous sand as a foundation material for island construction projects effectively reduces transportation costs and minimizes the reliance on expensive imported materials [11]. It is believed that the reef island airport with calcareous sand subgrade runway pavement structure is vulnerable to cluster munitions, whereby the projectile penetrate to a certain depth with detonation, as shown in Figure 1. The first stage damage composed of penetration is critical for the second stage internal explosion, which needs to be comprehensively studied for damage evaluation and munition optimization. In recent decades, extensive research has been conducted on the damage caused by various weapons to airport runways [12]. However, currently, the use of cluster munitions to create multiple craters on the runways, achieving the blockade of airports, has become the primary attacking strategy [13]. Based on the analysis of the crater characterization, and the mechanism of implosion in the runways obtained from the experiments and simulations, the damage modes of implosion in airfield runways can be classified into three categories: open crater mode, bulge crater mode, and hidden crater mode [14], as shown in Figure 1. In particular, for anti-runway munitions, the bulge crater, caused by explosion near interface, is the severest damage of the above three destruction modes, creating a relatively long blockade of the runway and being the most difficult and time-consuming to repair. Hence, the critical striking velocity corresponding to the interface location DOP is deemed to be the optimal penetration scenario. The gap between the literature and this work lies in the fact that the few attempts have been made to study reef island runway penetration. The critical striking velocity of projectile to penetrate to the interface of pavement and subgrade has not been reported yet. The purpose of this study aggregates to the state-of-the-art of the reef island runway pavement damage is to optimize the striking velocity to realize the proper penetration depth for the subsequent internal explosion. Such novelty not only benefits the cluster munitions attack strategy, but also promotes runway structure protection design.

The destructive effect and impact resistance performance of airport runways structured with multi-layer pavements under impacting loads have been a significant area of research. Han et al. conducted the field penetration and blast tests on airport pavements subjected to blast loading from a cluster bomb unit and assessed the condition of damage by numerical simulation, suggesting the rapid hardening high-strength concrete for the damaged concrete pavements repair [15]. Wu et al. developed a new muti-layer pavement system consisting of asphalt concrete layer reinforced with geogrid, high strength concrete layer, and engineered cementitious composites layer, and the system was compared with conventional concrete pavements through field blast tests and numerical modeling, revealing that the new multi-layer pavement had better blast resistance [3]. Zha et al. used the finite element simulation to compare the damage contours and dynamic responses of the anti-blasting asphalt pavement with a reinforced concrete layer and the conventional asphalt pavement, which showed that the additional reinforced concrete layer improved the blast resistance performance by about 20% [16]. To investigate the damage of scaled-down target of the airport runway, Wei et al. carried out experiment and numerical simulation to analyze the destructive effect of static explosion of charge after penetration and explosion of prefabricated hole, which showed that the crater size produced by the former explosion mode was obviously larger than that produced by the latter under the same explosion depth and explosive charge [17]. A three-dimensional graphics processing unit (GPU)-accelerated smoothed particle hydrodynamics (SPH) method was developed by Chen et al. [18], and was applied to the simulation of soil fragmentation and fracture propagation of the concrete-soil multilayered medium subjected to underground explosion involving millions of particles [19]. Comparing with the experimental data, the SPH model was able to reproduce the damage pattern of concrete slabs with different depths of explosives burial. Although the dynamic impact responses of airport runway with multi-layer pavements have been extensively studied through experiments and numerical simulation, there is still a knowledge gap in the research on pavement structures with calcareous sand as the subgrade, which hinders the development of island infrastructure engineering.

As the pavement structure surface layer, the mechanical properties of concrete have attracted wide attention [20], and the dynamic mechanical response of concrete panel or plate under the impactive loading conditions has been extensively studied [21]. Feng et al. obtained data on penetration depth, crater size, and net axial resistance through ballistic penetration tests and subsequent static deep indentation tests, and conducted numerical simulations using lattice discrete particle model [22]. Similarly, Liu et al. conducted experimental research and theoretical analysis on projectile penetration in concrete targets, examining destruction parameters such as ballistic depth and crater area [23]. The experiments and numerical simulations of the structural behavior of full-scale reinforced concrete slabs under blast loading were conducted by Castedo et al. [24], finding that the addition of steel fibers or polypropylene fibers to concrete slabs could effectively improve the tensile strength and blast resistance compared to simple reinforced concrete slabs. Feng et al. introduced the impact resistance theory to establish a semi-empirical analytical model for projectile perforation on steel–concrete–steel sandwich panels and an analytical model for perforation on steel–concrete panels, respectively, to predict the residual velocities and the target damage patterns [25], analyzing the dynamic response of the projectile on concrete–steel panels of different thicknesses [26]. The fiber reinforced concrete runway pavement proposed by Ali et al. [27] proved much better than the normal concrete pavement, and the researchers came up with finite element model to analyze the performance of improved concrete runway pavement under impact loading based on the results of drop weight impact test. However, adding fibers is not the sole method for enhancing the impact resistance of airport runways. Rubberized concrete also offered high impact resistance, and increased the ductility of runway pavements, as highlighted by Ferretti and Bignozzi [28]. Hardened steel ovoid nose projectiles with 19 mm diameter and three caliber-radius-head of 0.5 and 1 kg mass and 11.8 and 23.7 length-to-diameter ratios were used to impact the plain and reinforced concrete targets, in conjunction with numerical simulations in order to find out the behavior of the concrete against the impact loading, as well as to explore the length-to-diameter ratio of the projectile effect on the ballistic performance [29].

The Discrete Element Method (DEM) has been widely adopted to quantitatively analyze the deformation and damage processes occurring in concrete or geotechnical materials under both macro and micro mechanical regimes [30]. The unique advantage of DEM is that it is independent of numerical mesh, and allows large deformation and fracture of particles from the microscopic layer without deformation constraints [31], which is suitable for dealing with discontinuous problems [32]. Geng et al. applied DEM to analyze the infiltration distribution changes of tailing slopes and tailing landslides in different rainy seasons by constructing discrete units with specific elastic modulus and strength properties with the combination of the conversion formulas for the discrete units and numerical simulation experiments, realizing the automatic modeling of the discrete units [33]. Mechtcherine and Shyshko presented a numerical approach based on DEM to establish a link between the yield stress of the simulated concrete and the model parameters as a parametric reference for modeling the behavior of fresh concrete in different working processes, which showed that the numerical analysis agreed well with the final shape of the concrete in the slump-flow test [34]. DEM can also be used to study the physical process of impact-induced rock fragmentation in rockfall analysis [35]. A normal impact fragmentation model of synthetic spherical rock block under different impact loading rates was carried out by Shen et al. [36], which could reproduce the whole process of rock fragmentation under the impact, being in good agreement with the experimental observations. Huan et al. used matDEM1.60 software to develop an asteroid impact sampling model under microgravity conditions to explore the effects of the initial impact velocity and projectile shape on the internal characteristics of regolith and ejecta after impact, verifying the feasibility of discrete element simulation of the impact process [37]. The Lattice Discrete Particle Model (LDPM) was proposed to simulate concrete at the aggregate level and characterize the formation and evolution of cracks in concrete under loading, investigating the structural size and geometry effect on the strength and fracture process of concrete, which was proved to have a good agreement with experimental data and performed well in the prediction of splitting test results [38]. Therefore, it is advisable to apply DEM to model and investigate the dynamic responses of the airport runways with concrete and calcareous sand layers with matDEM as an effective tool.

In order to assess the damage effects of reef island airport runway pavements under projectile penetration, this paper adopted DEM simulation to numerically model the penetration of rigid projectiles into runway pavements with calcareous sand subgrade. The material parameters were calibrated with uniaxial compression and triaxial compression tests for subsequent penetration model validation. Furthermore, the penetration depth of airport runway structures with concrete pavement and calcareous sand base were numerically evaluated. Combining with back-propagation (BP) neural network and genetic algorithm, the critical velocity of airport runway penetration was predicted via intelligent optimization of pavement penetration. The results may shed some light on damage assessment, as well as anti-airfield runway cluster munitions design.

## 2. Discrete Modeling of Granular Materials

### 2.1. Discrete Element Method

**Figure 2:**
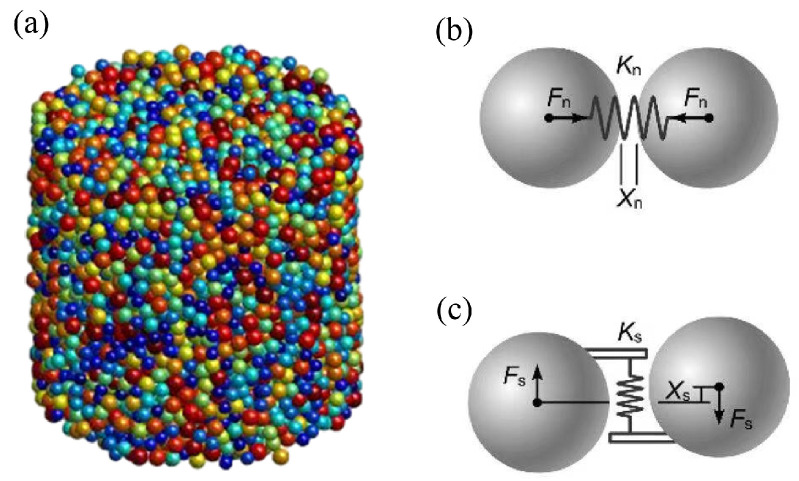
A 3D discrete element model with normal and shear spring force. (**a**) Stacking discrete particles. (**b**) Normal connection. (**c**) Shear connection.

In this study, a DEM model of granular material was established using MatDEM, achieving by stacking and cementing a large number of particles (Figure 2a) that conformed to Newtonian equations of motion, while the contact and interaction between the particles were simulated by a breakable elastic spring that acted only on the contact points between neighboring particles [30,39]. The forces between particles can be categorized into normal and tangential forces.

Figure 2b depicts the connection of two particles in the normal direction, including the normal force and normal deformation, which can be simulated by a normal spring. When the neighboring particles are connected to each other, the tensile or compressive spring force acting on the particle can be expressed as Equation (1).

(1) $$F_{n}=K_{n}\cdot X_{n},X_{n}<X_{b}$$

where $K_{n}$ is the normal stiffness, and $X_{n}$ is the normal displacement relative to the equilibrium position, denoting $F_{n}<0$ in compression and $F_{n}>0$ in tension.

When the normal relative displacement $X_{n}$ between the two particles is greater than the breaking displacement $X_{b}$, the normal spring between the particles breaks and the tension force becomes non-existent, at which time the normal spring force between the particles is zero. Under intact bonding conditions, the maximum normal force between the particles $F_{\mathrm{nmax}}$ can be calculated as Equation (2).

(2) $$F_{\mathrm{nmax}}=K_{n}\cdot X_{b}$$

where $X_{b}$ is the fracture displacement.

When the state of the two particles is in compressive contact, repulsive force is generated between them. The normal spring force can be expressed by Equation (3), and is negative.

(3) $$F_{n}=K_{n}\cdot X_{n},X_{n}<0$$

Similarly, the shear force and shear deformation between particles can be simulated by a tangential spring [40], as shown in Figure 2c, and the expression for the shear force ($F_{s}$) is expressed as Equation (4).

(4) $$F_{s}=K_{s}\cdot X_{s}$$

where $K_{s}$ is the shear stiffness, and $X_{s}$ is the tangential relative displacement.

The destruction of tangential springs follows the Mohr–Coulomb criterion, which is able to determine the maximum shear force of the intact bond, as expressed by Equation (5).

(5) $$F_{\mathrm{smax}}=F_{s0}-\mu_{p}\cdot F_{n}$$

where $F_{\mathrm{smax}}$ is the maximum shear force, $F_{s0}$ is the inter-particle shear force, and $\mu_{p}$ is the inter-particle coefficient of friction.

When $F_{n}$ is zero, $F_{\mathrm{smax}}$ reaches the maximum when only tensile contacts are considered, while the shear force increases with the increasing of the absolute value of the $F_{n}$ in compressive contact. When the external force exceeds the maximum shear, the tangential connection breaks and only sliding friction exists between the particles, as follows Equation (6).

(6) $$F_{\mathrm{smax}’}=-\mu_{p}\cdot F_{n}$$

### 2.2. Calibration for Concrete and Calcareous Sand

Mesoscopic mechanical parameters of DEM, including normal stiffness ($K_{n}$), shear stiffness ($K_{s}$), breaking displacement ($X_{b}$), initial shear resistance ($F_{s0}$), and friction coefficient ($\mu_{p}$), can be roughly estimated from five basic macroscopic mechanical properties theoretically, including Young’s modulus (*E*), Poisson’s ratio (ν), compressive strength ($f_{c}$), tensile strength ($f_{t}$), and internal friction coefficient (μ). The mechanical properties of elements are determined by the conversion formulas. In the regular packing model, there are analytical solutions between the micro mechanical parameters of the elements and the macro mechanical properties of the model, i.e., the conversion formulas of $K_{n}$, $K_{s}$, $X_{b}$, $F_{s0}$ and $\mu_{p}$ are written as Equations (7), (8), (9), (10) and (11), respectively.

(7) $$K_{n}=\frac{\sqrt{2}Ed}{4(1-2\nu )}$$

(8) $$K_{s}=K_{n}\frac{\left(1-5\nu \right)}{\left(1+\nu \right)}$$

(9) $$X_{b}=\frac{3K_{n}+K_{s}}{6\sqrt{2}K_{n}\left(K_{n}+K_{s}\right)}f_{t}d^{2}$$

(10) $$F_{s0}=\frac{1-\sqrt{2}\mu }{6}f_{c}d^{2}$$

(11) $$\mu_{p}=\frac{-2\sqrt{2}+\sqrt{2}I}{2+2I}$$

where *I* is the frictional parameter defined as $I={\left[{\left(1+\mu^{2}\right)}^{1/2}+\mu \right]}^{2}$.

In this section, uniaxial and triaxial compression tests were conducted on concrete and calcareous sand to calibrate the discrete element method parameters, thereby enhancing the credibility of the research on penetration resistance. The uniaxial compressive strength is a crucial indicator, reflecting the strength limit and stress–strain relationship under loading conditions, and is essential for analyzing the bearing capacity and deformation characteristics of concrete structures.

According to Table 1, the density, Young’s modulus, Poisson’s ratio, tensile strength, and compressive strength of concrete and calcareous sand were used as inputs for generating DEM particles. The concrete primarily consisted of coarse aggregate with a particle size of approximately 10 mm and fine aggregate with a particle size of about 1 mm. In contrast, calcareous sand had an irregular particle size, with an average ranging from 0.5 mm to 0.9 mm. The DEM parameters for the concrete shown in Table 2, under the condition of 23 MPa for a cubic specimen, are as follows: normal stiffness $K_{n}=5.42\times 10^{9}$ N/m, shear stiffness $K_{s}=5.38\times 10^{8}$ N/m, fracture displacement $X_{b}=3.76\times 10^{-7}$ m, initial shear resistance $F_{s0}=6.31\times 10^{4}$ N, and coefficient of friction $\mu_{p}$ = 0.19. For the calcareous sand sample, the required DEM parameters are: normal stiffness $K_{n}=1.01\times 10^{9}$ N/m, shear stiffness $K_{s}=1.14\times 10^{8}$ N/m, fracture displacement $X_{b}=3.02\times 10^{-5}$ m, initial shear resistance $F_{s0}=2.87\times 10^{4}$ N, and coefficient of friction $\mu_{p}$ = 0.14.

**Figure 3:**
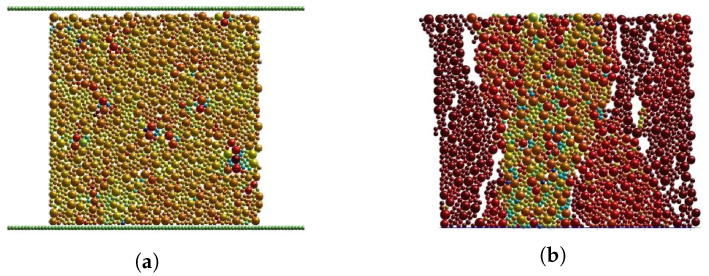
DEM parameter calibration for concrete. (**a**) Uniaxial compression. (**b**) Fracture under compression.

**Figure 4:**
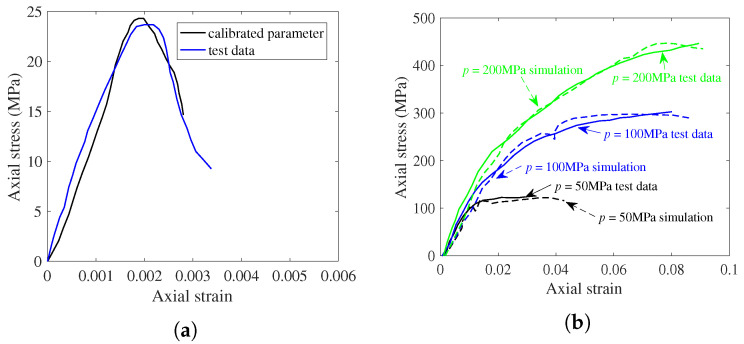
DEM parameter calibration for concrete. (**a**) Uniaxial compression response. (**b**) Triaxial compression response.

Based on the experimental data obtained by Warren et al. [41], numerical simulation of uniaxial compression was carried out for 15 cm × 15 cm × 15 cm cubic concrete specimens. Fixed constraint was conducted at the bottom of the specimen and the load was applied at the top at a rate of 2.4 N/ms [42]. The loading condition and the final vertical fracture damage of the model are illustrated in Figure 3. The simulated stress–strain curve behaved consistently with the curve obtained from the uniaxial compression test, as shown in Figure 4a. Initially, the concrete worked in the elastic stage, and the stress increased proportionally with the strain. With the increase in compressive stress, micro-cracks inside the concrete gradually developed and plastic strain began to occur, the stress–strain curve no longer maintained a linear relationship, and the slope gradually decreased. Upon reaching the peak stress, visible cracks began to appear on the surface of the specimen, and under continued loading, these cracks rapidly penetrated from the surface through the interior of the material, leading to complete destruction of the specimen.

**Figure 5:**
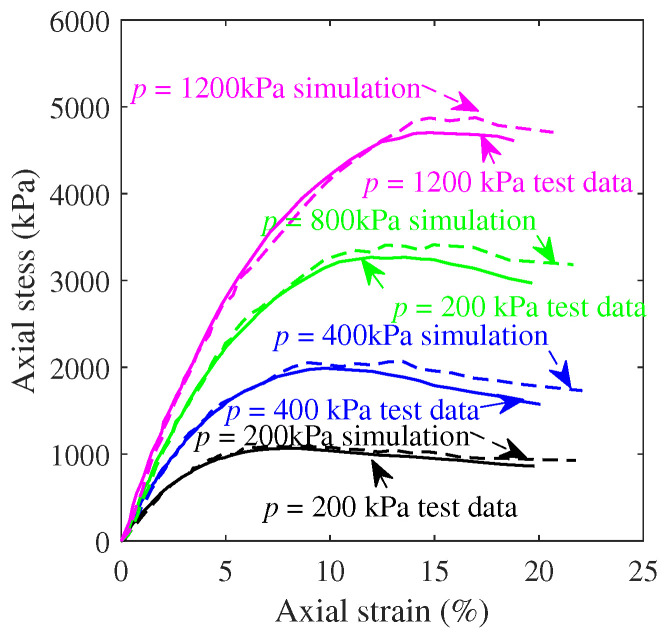
Calibrated model of calcareous triaxial compression.

To assess the mechanical properties and strength characteristics of concrete and calcareous sand under more complex stress states, triaxial compressive responses were conducted using axial displacement control in numerical simulations while maintaining constant lateral confining pressure. Figure 4b displays the axial stress versus axial strain curves for concrete under limiting pressures of 50 MPa, 100 MPa, and 200 MPa. Figure 5 shows the corresponding curves for calcareous sand at limiting pressures of 200 kPa, 400 kPa, 800 kPa, and 1200 kPa. The stress–strain curves of calcareous sands exhibited nonlinearities, which were mainly caused by particle crushing and the transformation of the relative positions of sand particles during the loading process [43]. The curves of both calcareous sand and concrete had the similar variabilities, which showed that the longitudinal strength and deformability of the specimens of the two materials improved with the increase in transverse pressure. The peak values of the stress–strain curves rose, and the decline trend after the maximum stress value gradually slowed down with the increase in the ultimate pressure value, while the peak stress, peak strain, and elastic modulus also increased significantly. Additionally, the increase in lateral pressure restricted the transverse deformation of the specimens, thereby limiting both lateral expansion and the propagation of internal microcracks. Strain hardening arose from the heterogeneity of the granular skeleton of concrete and calcareous sand materials [44], and the axial response exhibited a transition from strain softening to strain hardening as the microstructure within the materials changed with the increasing confining pressure [45].

### 2.3. Validation of Penetration Model

The validation of the discrete element method in simulating uniaxial and triaxial compression tests for concrete and calcareous sand has been validated in the previous section, demonstrating its potential for more complex loading simulations of these materials. In this section, the numerical performance of DEM for the penetration modeling of concrete and calcareous sand were validated by both the depth of penetration and projectile deceleration.

**Figure 6:**
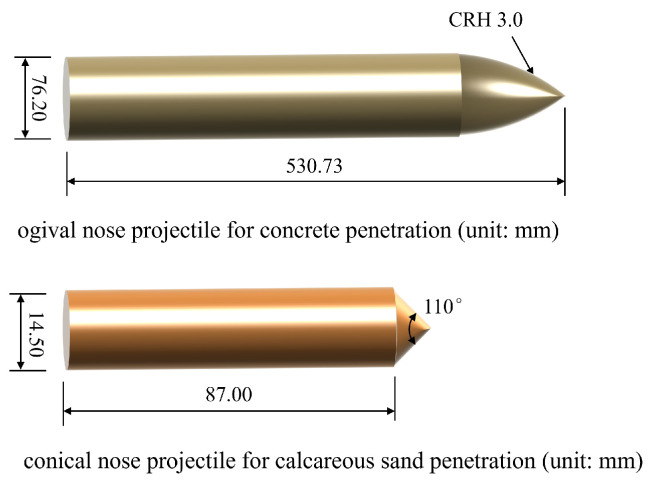
Dimension of projectiles for penetration simulation validation.

As dimensioned in Figure 6, the ogival nose projectile for concrete penetration with 76.2 mm caliber, 3.0 CRH, and a total length of 530.73 mm was machined from 4340 RC45 steel, and the nominal mass of the projectile was 13 kg. The conical nose projectile for calcareous sand was internally hollow, with a diameter of 14.5 mm and a length of 87 mm, which was made of high-strength alloy steel with a density of 7850 kg/m^3^, as mentioned by Miao et al. [46]. The body of projectile was set to be a rigid material due to the negligible wear and tear during the penetration process.

The concrete penetration target was cylindrical with a diameter of 1.83 m, which was large enough to avoid rear and lateral boundary effect, and meanwhile, the target was laterally constrained. The calcareous sand penetration target had a cross-sectional size of 0.6 × 0.6 m and a thickness of 1.7 m, with fixed lateral boundaries. Penalty contact was adopted to compute the normal contact force between the granular material particles and the rigid projectile [47]. The experimental data for penetration into concrete and calcareous sand, as modeled in this paper, were derived from Forrestal [48] and Miao [46], respectively. The numerical results of DOP values for both concrete and calcareous sand under impact tests are presented in Table 3 and Table 4, showing that penetration into concrete or calcareous sand was validated by examining the DOP, with a maximum relative error not greater than 5.6%.

**Figure 7:**
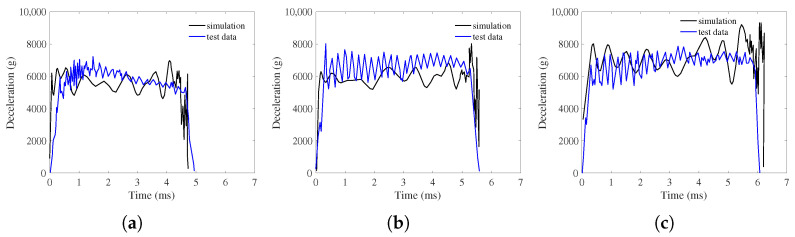
Simulation and test deceleration curves of penetration for concrete with different striking velocities. (**a**) 238 m/s striking velocity. (**b**) 276 m/s striking velocity. (**c**) 370 m/s striking velocity.

**Figure 8:**
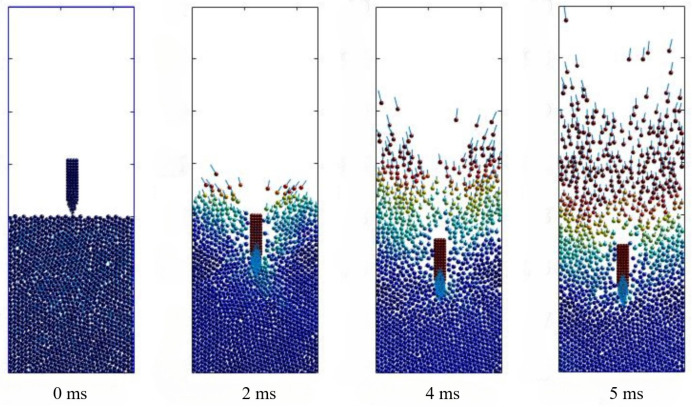
Particle velocity contour of concrete penetration simulation.

The deceleration of simulations and tests for projectiles penetrating concrete targets at three different striking velocities of 238 m/s, 276 m/s, and 370 m/s were comparatively presented in Figure 7, with g denoting the gravitational acceleration. In terms of general trends, the DEM simulation results matched well with the experimental deceleration curves. The deceleration histories for all striking velocities exhibited a rapid increase during the initial projectile nose penetration phase followed by a nearly flat plateau corresponding to the tunneling phase, where the deceleration values remained relatively steady but fluctuated slightly due to the penalty function contact model between the concrete particles and the projectile. Additionally, higher impact velocities extended the duration of the tunneling phase and resulted in higher deceleration peak values. At last, the projectile suffered with increasing resistance inside the target, causing a sharp drop in deceleration to zero after the plateau phase. The cross sections views of the simulation of projectile penetration into a concrete target at different moments are illustrated in Figure 8, where the depth of penetration gradually increased with time. The concrete particles were pushed away by the projectile nose, which were squeezed to cause concrete scattering fragments around the crater.

**Figure 9:**
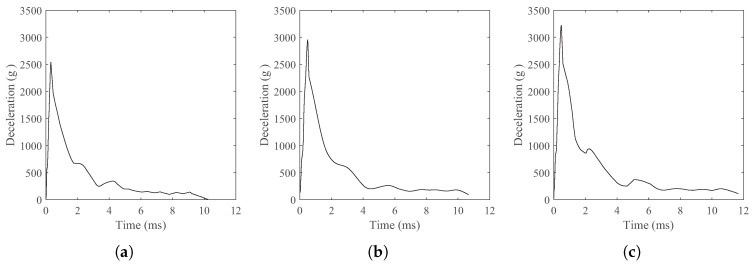
Simulation deceleration curves of penetration for calcareous sand with different striking velocities. (**a**) 411 m/s striking velocity. (**b**) 583 m/s striking velocity. (**c**) 710 m/s striking velocity.

Obtained from the numerical simulations, the projectile deceleration histories during calcareous sand penetration are plotted in Figure 9. In the early stage of the projectile penetration, the deceleration increased rapidly in a short period of time until the maximum value, which was affected by the velocity. The higher striking velocities resulted in higher peak deceleration, which was about 32% higher at 710 m/s compared with that at 411 m/s. After the peak point, the deceleration of the projectile declined rapidly, and subsequently entered a platform phase. At this time, the target static resistance played a dominant role in the penetration resistance, decreasing the deceleration slowly [49].

## 3. Results of Pavement Structure Penetration Simulations

Given the specific characteristics of the reef island airport runway, the penetration process is divided into two distinct phases: the concrete penetration phase and the calcareous sand penetration phase. In this section, the previously validated DEM model was employed to conduct numerical simulations of runway pavement penetration with various striking velocities and concrete thicknesses, with the aim of providing a comprehensive assessment of the airport runway structure under impact.

**Figure 10:**
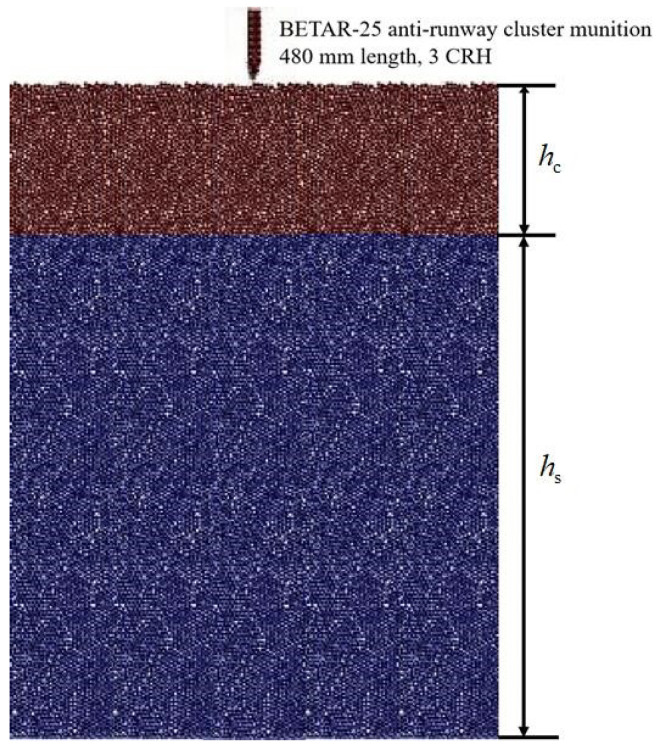
Simulation model of projectile penetration into pavement structure.

The model of projectile penetration into airport runway pavement structure is depicted as Figure 10. In this paper, the Russian-made BETAR-25 anti-runway cluster munition was selected for numerical simulation, with ogival nose projectile body with a total weight of 9–13 kg, a diameter of 76 mm, 3.0 CRH, and an overall length of 480 mm [50]. With striking velocity of 170 m/s–280 m/s, the projectile is supposed to impact the target with normal penetration.

The reef island airport runway model was constructed as a two-layer composite structure with a concrete layer $h_{c}$ for the surface and a calcareous sand layer $h_{s}$ for the base, measuring 1.8 m × 1.8 m area, with lateral constraints. The model particles were randomly generated using Fuller’s coefficient 0.5 in the range of aggregate particle size of 5–15 mm.

**Figure 11:**
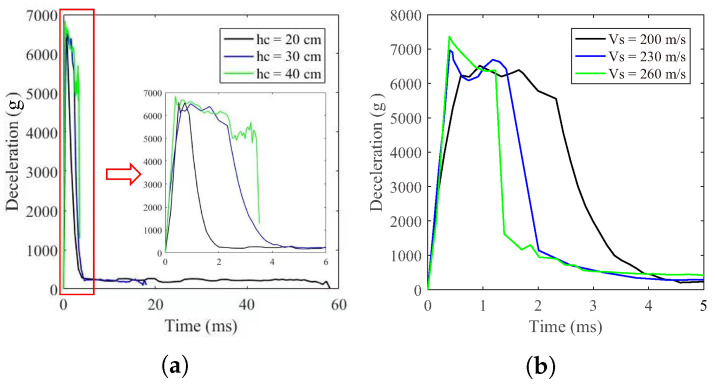
Projectile deceleration during penetration into runway pavement structure. (**a**) Various concrete layer thickness. (**b**) Various projectile striking velocities.

Table 5 shows the simulation results of DOP of various velocities of projectile and thickness of concrete layer. Among all of the results, three representative striking velocities (200 m/s, 230 m/s and 260 m/s), as well as three different concrete thickness (20 cm, 30 cm, 40 cm), have been selected and plotted in Figure 11 with three different colors. During the surface concrete layer penetration phase, the velocity of the projectile into the concrete decreased while the deceleration initially increased. As the resistance reached its maximum value, the deceleration also peaked and was maintained in a short plateau period with slight fluctuations. However, as the projectile penetrated deeper, it was subjected to the resistance, leading to the decline of velocities and decelerations. After entering the calcareous sand layer, which offered significantly lower resistance than concrete, the reduction in velocities slowed down. In addition, when the projectile navigated through the pit-opening stage in the calcareous sand layer, the deceleration rose slightly, and after the pit-opening stage, the resistance acting on the projectile head became smaller due to the degradation of the tensile stress between the surface of the projectile body and the calcareous sand particles [45]. On the other hand, the lower striking velocities further reduced the resistant force. These two main factors both contribute to the decrease in the deceleration at a low rate and the stabilization with slight fluctuations.

Since the impact velocity and mass of the projectile were constant, the peak of the deceleration curves remained almost unchanged, regardless of the target thickness, although thicker targets led to longer penetration durations. However, the projectile was unable to penetrate the concrete layer at a thickness of 40 cm. Variations in striking velocities resulted in changes to peak deceleration, with higher velocities producing greater peak deceleration values and shorter penetration durations.

## 4. Intelligent Optimization Results of Pavement Penetration

### 4.1. GABP Neural Network Proxy Model

**Figure 12:**
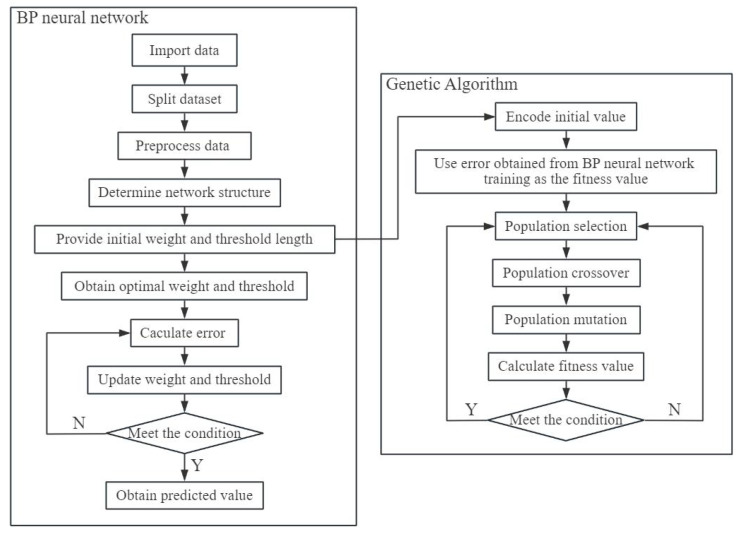
Flowchart of GABP proxy model.

The back propagation neural network (BPNN) is a multi-layer feedforward network that employs the error backpropagation algorithm [51], which is considered one of the most classic and extensively utilized models in neural networks [52,53]. BPNN excels in handling non-linear relationships, and in the training process of continuous iteration, the primary concept involves computing the error between the output data and the expected data, followed by adjusting the weights and thresholds in a retrograde fashion to minimize the error [54,55]. The conventional BP neural network algorithm relies on the gradient descent technique for optimization. However, the random initialization of weights and thresholds, coupled with the issue of wide variation in initial value selection, may lead the network to become stuck in local optima [56]. To improve the optimization efficiency of pavement penetration, this work adopted a novel proxy model approach, combining genetic algorithms with (GA) neural network models, improving the forecasting capabilities of the overall model. The GABP proxy model flowchart is shown in Figure 12, implying the following detailed steps.

Step 1: Data preprocessing and network structure determination. Divide the original data into training, testing, and validation sets, adopt the single hidden layer BPNN model, and configuring genetic algorithm parameters.

Step 2: Population initialization and interactions. Encode the initial value according to the fitness function and generate new individuals by carrying out selection, crossover, and mutation operations.

Step 3: New population updating. Recalculate the fitness value for the updated group, update the global optimal individual meeting the fitness value conditions until reaching the maximum number of iterations.

Step 4: BP neural network training. Assign the optimal individual for BPNN, obtain the optimal weights and thresholds, perform training simulations on error evaluation calculations and backpropagation until convergence.

#### 4.1.1. Data Processing

The airport runway penetration scenarios were obtained from a series of simulations, encompassing projectile weight, pavement concrete thickness, striking velocity, and penetration depth as the features. A total of 52 samples were gathered, which are shown in Table 5, with 80% of the data randomly assigned to the training set and the rest allocated to the testing set. To convert input and output data to values within the range of [0, 1], the transformation formula is utilized as Equation (12).

(12) $${\bar{R}}_{i}=\frac{R_{i}-R_{\min}}{R_{\max}-R_{\min}}$$

where $R_{i}$, $R_{\min}$, $R_{\max}$ represent the input data, the minimum value, and the maximum of the data, respectively.

#### 4.1.2. Development of GABP Proxy Model

**Figure 13:**
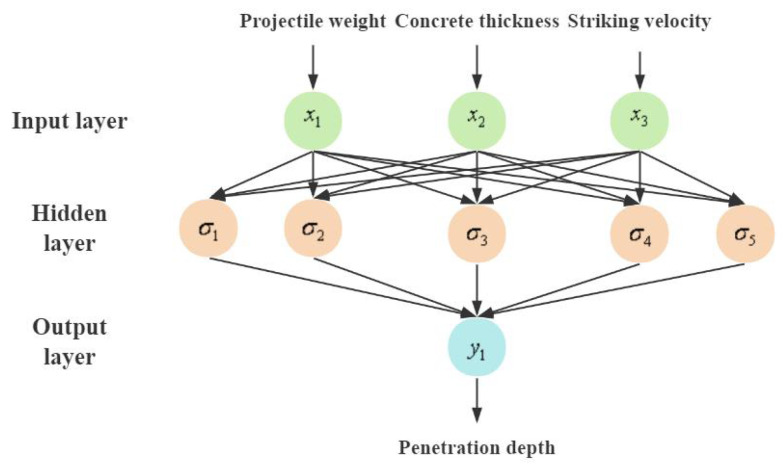
Structure of BPNN.

The BP neural network structure is shown in Figure 13. The input layer comprised three nodes corresponding to three eigenvectors: projectile weight, pavement concrete thickness, and striking velocity. The output layer consisted of one node representing penetration depth. The number of nodes in the hidden layer was determined by the neurons in the input and output layers, with five neurons chosen for the hidden layer in this study.

The initial parameters were configured as 1000 times training iterations with a target of 0.005 and a learning rate set to 0.1 using the TRAINLM training function. In the genetic algorithm, 500 evolutions were performed with a population size of 200. The selection factor was set to 0.09, while the crossover factor was randomly chosen within the range of 0 to 1. A non-uniform mutation approach was applied, utilizing a non-uniform probability distribution to adjust parent parameters. This implies that the mutation probability evolved over time, potentially decreasing as iterations increased or as the population included a greater number of fitter individuals. The initial parameters for the GABP proxy model are presented in Table 6.

#### 4.1.3. Assessment of GABP Proxy Model

In order to evaluate the accuracy of the training and prediction performance, two statistical parameters, which are the root mean square error (RMSE) and determination coefficient ($R^{2}$), are served as the error discrimination criteria [57]. The root mean square error (RMSE) indicates the predictive capability of the model while determination coefficient assesses the fit of the model to the data. The definitions for RMSE and $R^{2}$ are shown as Equations (13) and (14).

(13) $$RMSE=\sqrt{\frac{1}{n}\sum_{i=1}^{n}{\left(P_{i}-R_{i}\right)}^{2}}$$

(14) $$R^{2}=1-\frac{\sum_{i=1}^{n}{\left(P_{i}-R_{i}\right)}^{2}}{\sum_{i=1}^{n}{\left(\bar{R}-R_{i}\right)}^{2}}$$

where *n* is the number of samples, $P_{i}$ is the predicted value, $R_{i}$ is the real experimental value, and $\bar{R}$ is the average of the real values. The smaller RMSE value indicates better predictive ability of the model; similarly, the value of $R^{2}$ closer to 1 indicates a better fitting effect of the model.

**Figure 14:**
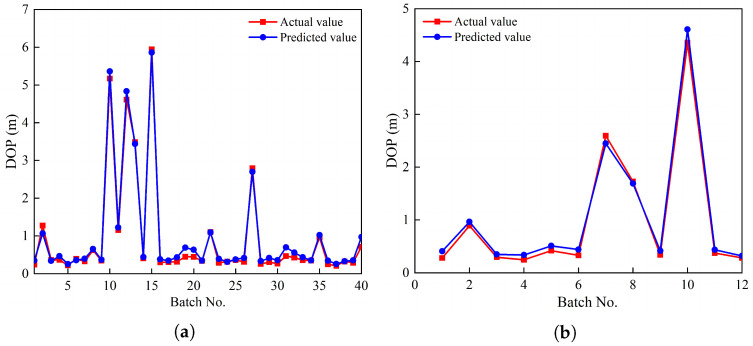
Output of model data. (**a**) Training data. (**b**) Testing data.

Figure 14 displays the scatter plot of predicted versus actual values for both networks, which are closely related to each other. The GABP neural network proxy model performance peaked at epoch 10, with a regression correlation coefficient exceeding 0.99 across all sets. The average error of the training and testing sets shown in Table 7 are 0.0886 m and 0.0971 m. The subsequent prediction results demonstrated that the BP neural network optimized by the GA algorithm not only significantly validated the accuracy of the neural network model, but also exhibited superior performance in predicting the airport runway penetration depth.

### 4.2. Genetic Algorithm Search for Critical Striking Velocity

Based on crater morphology and crack extension, the damage patterns of airport runways can be classified into three categories: open crater, bulge, and hidden crater patterns [14], as illustrated in Figure 1. From the perspective of post-damage repair, the open crater and hidden crater damage modes are generally considered easier to repair. In wartime emergencies, these types of damage can often be addressed by simply clearing and filling the damaged area, followed by the placement of a steel plate to allow temporary aircraft operations. However, when rumble damage occurs in the surface layer concrete, the presence of ring cracks penetrating the surface layer significantly increases the volume of material to be removed, making repairs much more challenging. Consequently, genetic algorithms have been used to predict the critical velocity at which concrete layer is just perforated, in order to maximize explosion damage to the pavement structure.

A genetic algorithm is a computational method that randomly searches for optimal solutions by simulating the natural evolutionary process [58,59], which draws on natural selection and genetic mechanisms in biology. The genetic algorithm mainly includes population initialization, fitness function evaluation, and genetic operator determination. The fitness evaluation function reflects the ability of individuals to adapt to the environment. Genetic operators mainly include selection, crossover and mutation [60]. Through the iterative repetition of these steps, the algorithm gradually searches for the optimal solution.

The initial population size was set to 100, with a maximum of 100 iterations and the crossover probability and mutation probability were set at 0.8 and 0.2, respectively. The optimal penetration velocity at the point of maximum damage was determined using the condition of just penetrating the concrete surface as the constraint boundary. Additionally, the larger adaptation degree represented the optimal penetration speed corresponding to it.

**Figure 15:**
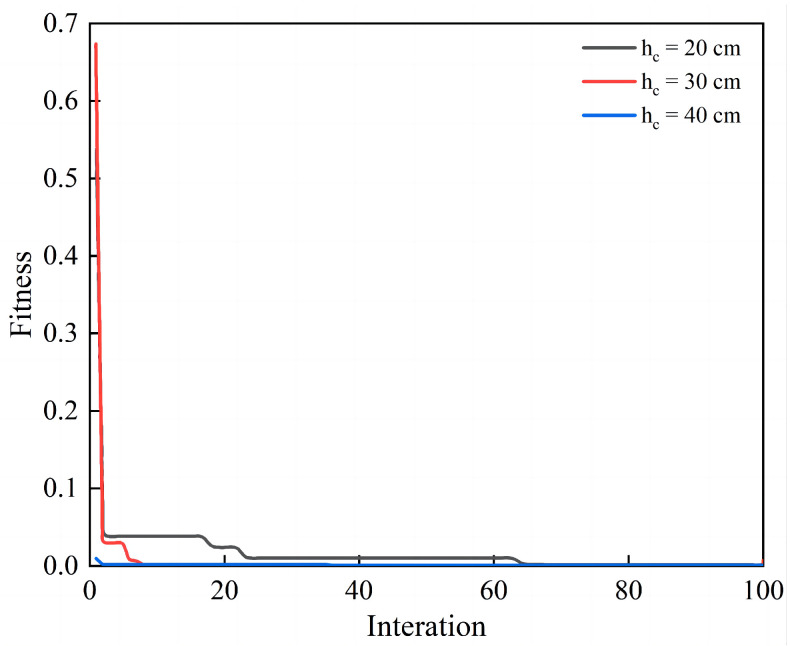
The convergence graph of GA.

As can be seen from Figure 15, we used the fitness function as a criterion for the loss value. Observing the Loss value, the concrete layer of 20 cm had converged in the twenty-second iteration, the concrete layer of 30 cm had converged in the eighth iteration, and the concrete layer of 40 cm had converged in the third iteration. The fitness function proved that the GA neural network proxy model provided a good fit and prediction of the actual values and the model had a strong explanatory power. When the thickness of the concrete surface layer was 20 cm, 25 cm, 30 cm, 35 cm, and 40 cm, the optimal speed was 282.19 m/s, 308.79 m/s, 328.68 m/s, 345.34 m/s, and 378.39 m/s, respectively, which is shown in Table 8.

## 5. Concluding Remarks

It is of great interest to assess the damage of the reef island airport pavement with multi-layer structures under impact loading. In this study, DEM was employed to model rigid projectile penetration through runway pavements composed of concrete and calcareous sand, with the model calibrated using uniaxial and triaxial compression tests. The critical impact velocity causing maximum explosion damage was determined using GA optimization via GABP proxy model. The main conclusions are drawn as follows.

(1) With mesoscale constitutive laws governing the interaction between adjacent particles, DEM parameters of concrete and calcareous sand granular materials could be calibrated by modeling features like cohesive fracture in uniaxial compression and pressure hardening in triaxial compression. The deep penetration models were validated against test data in terms of DOP with relative error ≤ 5.6%. This work provide a DEM-based simulation tool for concrete or calcareous sand granular penetration modeling during damage assessments.
(2) Deceleration history of projectile penetration in concrete pavement and calcareous sand subgrade multi-layer structure suggested a pulse up to 7000 g deceleration due to concrete layer perforation resistance, which is followed by a long tail resulting from low strength calcareous sand medium static resistance.
(3) The BP neural network optimized by the GA algorithm was capable of predicting the depth of penetration of airport runways pavement structures with high accuracy. The proposed GABP proxy model may help to realize rapid and robust DOP prediction during reef airport runways damage evaluations, as well as pavement structural design.
(4) Calling the GABP proxy model, the GA optimization was performed for most destructive effect of concrete surface layer with thickness of 20 cm, 25 cm, 30 cm, 35 cm, and 40 cm, whereby the critical velocities were predicted as 282.19 m/s, 308.79 m/s, 328.68 m/s, 345.34 m/s, and 378.39 m/s, respectively.

## Figures and Tables

**Table 1:** Macroscopic mechanical properties of concrete and calcareous sand.

Material	Density	Young’s Modulus	Poisson’s Ratio	Tensile Strength	Compressive Strength
Concrete	2.38 g/cm^3^	30 GPa	0.283	3 MPa	23 MPa
Calcareous sand	1.48 g/cm^3^	0.38 MPa	0.363	1 kPa	0.283 kPa

**Table 2:** Mesoscopic inter-particle mechanical parameters of concrete and calcareous sand for MatDEM.

Parameter	Concrete	Calcareous Sand
Normal stiffness, $K_{n}$ (N·$m^{-1}$)	5.42 × $10^{9}$	1.01 × $10^{9}$
Shear stiffness, $K_{s}$ (N·$m^{-1}$)	5.38 × $10^{8}$	1.14 × $10^{8}$
Fracture displacement, $X_{b}$ (m)	3.76 × $10^{-7}$	3.02 × $10^{-5}$
Initial shear resistance, $F_{s0}$ (N)	6.31 × $10^{4}$	2.87 × $10^{4}$
Coefficient of friction, $\mu_{p}$	0.19	0.14

**Table 3:** Discrete simulation prediction of concrete penetration depth (unit: m).

No.	Striking Velocity (m/s)	Experimental DOP [48]	Numerical DOP	Relative Error
1	250	0.62	0.59	5.6%
2	337	0.93	0.89	4.5%
3	370	1.18	1.15	3.0%

**Table 4:** Discrete simulation prediction of calcareous sand penetration depth (unit: m).

No.	Striking Velocity (m/s)	Experimental DOP [46]	Numerical DOP	Relative Error
4	411	1.06	1.01	4.7%
5	583	1.10	1.06	3.6%
6	710	1.23	1.19	3.3%

**Table 5:** Simulation results of depth of concrete penetration.

No.	$m_{p}$ (kg)	$V_{s}$ (m/s)	$h_{c}$ (cm)	DOP (m)	No.	$m_{p}$ (kg)	$V_{s}$ (m/s)	$h_{c}$ (cm)	DOP (m)
1	13	200	15	4.612	27	9	195	40	0.26
2	13	200	20	3.486	28	9	205	35	0.28
3	13	200	30	0.622	29	9	215	40	0.302
4	13	200	40	0.365	30	9	225	35	0.325
5	8	200	30	0.246	31	9	235	40	0.348
6	9	200	30	0.27	32	9	245	35	0.373
7	10	200	30	0.294	33	9	255	40	0.397
8	11	200	30	0.318	34	9	265	35	0.961
9	12	200	30	0.348	35	9	275	40	0.467
10	13	220	30	2.595	36	9	285	40	1.156
11	13	240	30	4.355	37	10	250	35	0.891
12	13	250	30	5.17	38	10	230	35	0.367
13	13	260	30	5.947	39	10	210	35	0.317
14	13	220	40	0.429	40	11	240	35	1.103
15	13	240	40	1.728	41	11	215	35	0.356
16	13	250	40	2.794	42	11	195	35	0.305
17	13	190	40	0.336	43	12	234	40	0.450
18	13	180	40	0.307	44	12	241	40	0.713
19	13	170	40	0.281	45	12	207	35	0.361
20	9	210	30	0.291	46	12	193	35	0.322
21	9	230	30	0.341	47	10	198	40	0.289
22	9	250	30	1.276	48	10	209	40	0.315
23	9	190	30	0.25	49	10	246	40	0.410
24	9	170	30	0.212	50	11	237	40	0.419
25	9	175	40	0.221	51	11	205	40	0.330
26	9	185	35	0.24	52	11	244	40	0.446

**Table 6:** Parameters of the GABP proxy model.

Parameters	Value
Neurons at the input layer	3
Neurons at the hidden layer	5
Neurons at the hidden layer	1
Learning rate	0.01
Total epochs	1000
Training function	Levenberg–Marquardt backpropagation (TRAINLM)
Population size	100
Number of evolutions	500
Selection factor	0.09
Crossover factor	Random number within [0, 1]
Mutation factor	Non-uniform mutation

**Table 7:** Precision parameter of the GABP proxy model.

Stage	Training	Testing
RMSE	0.1131	0.1124
$R^{2}$	0.9934	0.9916
Average error (*m*)	0.0886	0.0971

**Table 8:** Critical velocity of different thickness of concrete (unit: cm).

Thickness (cm)	Velocity (m/s)	Fitness
20	282.19	0.0025
25	308.79	0.0004
30	328.68	0.0001
35	345.34	0.0017
40	378.39	0.0063

## Data Availability

The data presented in this study are available on request from the corresponding author.
